# Algae-Derived Bioactives Reprogram the Gut–SIRT1–Kisspeptin Axis in Polycystic Ovary Syndrome

**DOI:** 10.3390/md24050185

**Published:** 2026-05-20

**Authors:** Arifa Mustika, Era Gorica, Dante Saksono Harbuwono, Eighty Mardiyan Kurniawati, Edwin Hadinata, Amal Arifi Hidayat, Salmon Charles Pardomuan Tua Siahaan, Hendy Hendarto, Antonello Santini, Fahrul Nurkolis

**Affiliations:** 1 Department of Anatomy, Histology, and Pharmacology, Faculty of Medicine, Universitas Airlangga, Surabaya 60131, Indonesia; 2 Department of Pharmacy, Faculty of Medical Sciences, Albanian University, 1017 Tirana, Albania; 3 Department of Cardiac Surgery, Faculty of Medicine, University Hospital Zürich and University of Zürich, Wagistrasse 12, 8952 Schlieren, Zurich, Switzerland; 4 Division of Endocrinology, Metabolism, and Diabetes, Department of Internal Medicine, Faculty of Medicine, Universitas Indonesia, Dr. Cipto Mangunkusumo National Referral Hospital, Jakarta 10430, Indonesia; 5 Department of Obstetrics and Gynecology, Faculty of Medicine, Universitas Airlangga, Surabaya 60132, Indonesia; 6 School of Medicine, Faculty of Medicine, Universitas Ciputra, Surabaya 60219, Indonesia; 7 Division of Gastroenterohepatology, Department of Internal Medicine, Faculty of Medicine, Universitas Airlangga, Surabaya 60132, Indonesia; 8 Medical Research Center of Indonesia, Surabaya 60281, Indonesia; fahrul.nurkolis.mail@gmail.com; 9 Department of Pharmacy, University of Napoli Federico II, Via Domenico Montesano, 49, 80131 Napoli, Italy; 10 Institute for Research and Community Service, State Islamic University of Sunan Kalijaga (UIN Sunan Kalijaga), Yogyakarta 55281, Indonesia; 11 Faculty of Medicine, Universitas Airlangga, Surabaya 60132, Indonesia

**Keywords:** polycystic ovary syndrom, gut microbiota, SIRT1 signaling, women’s health, algae-derived bioactives, kisspeptin, gut–brain axis, neuroendocrine regulation, metabolic dysfunction

## Abstract

Polycystic ovary syndrome (PCOS) is increasingly recognized as a complex, multi-system disorder involving interactions among metabolic dysfunction, chronic low-grade inflammation, and neuroendocrine dysregulation, rather than a condition confined to the ovary. While current management strategies primarily target symptomatic manifestations, such as menstrual irregularity, hyperandrogenism, and insulin resistance, they do not directly address the underlying integrative pathways linking the gut microbiome, cellular energy sensing, and hypothalamic reproductive control. This review proposes a mechanistic framework in which algae-derived bioactives modulate a gut–SIRT1–kisspeptin axis, thereby offering a systems-level perspective on PCOS pathophysiology and intervention. Gut dysbiosis in PCOS contributes to altered bile acid signaling, disrupted microbial metabolite profiles, and increased inflammatory tone, all of which may impair both metabolic and reproductive functions. Concurrently, reduced activity of the NAD^+^-dependent deacetylase SIRT1 has been documented across ovarian, endometrial, and metabolic tissues, linking energy imbalance to oxidative stress, inflammation, and impaired steroidogenesis. At the neuroendocrine level, dysregulated kisspeptin signaling contributes to abnormal gonadotropin-releasing hormone pulsatility and luteinizing hormone hypersecretion, key features of PCOS. Algae-derived compounds, including polysaccharides, phlorotannins, fucoidan, fucoxanthin, and microalgae bioactives, exhibit prebiotic, anti-inflammatory, and metabolic regulatory properties that intersect with these pathways, particularly through modulation of gut microbiota and activation of AMPK/SIRT1 signaling. The central proposition of this review is that algae-derived bioactives may act across interconnected biological layers: reshaping gut microbial ecology, restoring SIRT1-mediated metabolic balance, and retuning kisspeptin-driven neuroendocrine activity. While individual components of this axis are supported by substantial evidence, direct experimental validation of the complete pathway remains limited. Therefore, this framework is positioned as a translationally grounded but hypothesis-driven model that integrates currently fragmented findings into a coherent and testable paradigm. Future research should prioritize multi-level experimental and clinical studies that simultaneously assess microbiota composition, metabolic signaling, and reproductive neuroendocrine outcomes to establish the therapeutic potential of algae-based interventions in PCOS.

## 1. Introduction

**Figure 1:**
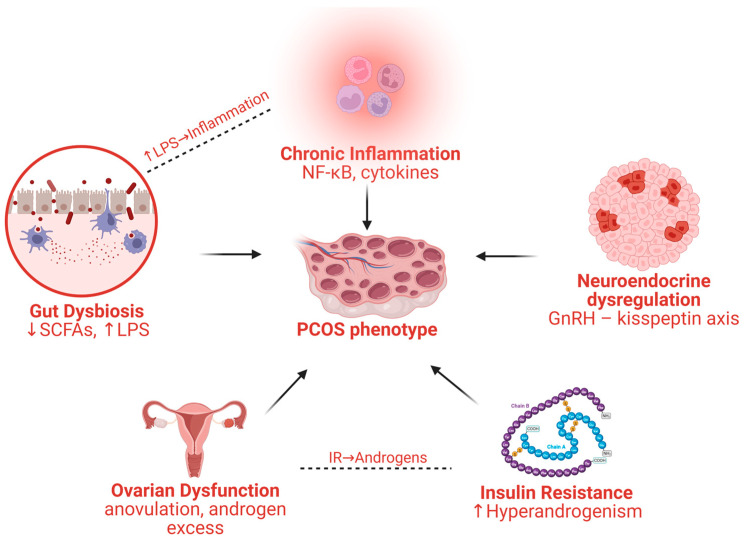
PCOS as a multi-axis system disorder.

Polycystic ovary syndrome (PCOS) is a heterogeneous syndrome defined clinically by combinations of ovulatory dysfunction, hyperandrogenism, and polycystic ovarian morphology (Figure 1), but its burden extends far beyond the ovary [1]. The 2023 international evidence-based guideline stresses that PCOS spans reproductive, metabolic, and psychological domains, remains under-recognized, and is still supported by an evidence base that is often only low to moderate quality [2]. In parallel, epidemiological work in adolescents shows how strongly prevalence estimates depend on diagnostic criteria: when polycystic ovarian morphology is omitted in adolescents, prevalence is materially lower than with older Rotterdam-based definitions [3], reinforcing that PCOS is not a single static entity but a syndrome whose expression changes with age, phenotype, and context.

Such clinical variability has important implications because it challenges organ-centered models of PCOS. Patients may present with markedly different dominant features, ranging from insulin resistance and central adiposity to hyperandrogenism, neuroendocrine disruption, or endometrial dysfunction. Such variability suggests that PCOS cannot be adequately explained as an isolated ovarian disorder, but rather as a condition arising from dynamic interactions among the gut, liver, adipose tissue, brain, ovary, and endometrium [4,5]. This systems-level perspective is increasingly supported by contemporary reviews integrating metabolic, neuroendocrine, and microbiome-related mechanisms [6]. These reviews converge on a common theme; PCOS behaves as a network disorder in which metabolic stress and abnormal neuroendocrine firing reinforce one another, rather than as a purely ovarian lesion.

The classical therapeutic arsenal remains clinically useful but mechanistically incomplete. Lifestyle intervention is recommended for all women with PCOS to improve metabolic health [7]. Combined oral contraceptives are recommended mainly for hirsutism and menstrual irregularity. Metformin is positioned mainly for metabolic outcomes and can be considered for cycle regulation in selected groups. Letrozole is the first-line pharmacological option for ovulation induction in anovulatory infertility [8]. Yet, these strategies chiefly target the visible phenotype at the point of care. They do not explicitly repair mucosal barrier failure, altered bile acid signaling, disturbed microbial metabolite flux, or hypothalamic pulse-generator instability. As a result, many patients experience partial benefit, not a mechanistic reset.

This gap is where algae-derived bioactives become conceptually powerful. Algae are not one intervention but a library of compounds with layered bioactivity. Brown seaweeds contribute fucoidan, alginate, laminarin, fucoxanthin, and phlorotannins [9]. Green microalgae such as *Chlorella vulgaris* offer fiber-like cell-wall material, pigments, carotenoids, peptides, and antioxidant micronutrients [10]. Cyanobacterial biomass such as *Spirulina platensis* provides phycocyanin-rich protein, polysaccharides, and lipid fractions with established metabolic activity [11]. Unlike single-target drugs, these materials are unusually suited to multi-axis disease because some fractions reach the colon and reshape microbial fermentation, while others act systemically on oxidative stress, mitochondrial function, insulin signaling, or steroidogenic pathways.

The central argument of this review is therefore not simply that “algae may help PCOS,” which would be too broad to be novel. The more precise proposition is that algae-derived compounds may reprogram a gut–SIRT1–kisspeptin axis that links three pathophysiological layers: microbial ecology, cell-intrinsic metabolic sensing, and hypothalamic control of gonadotropin secretion [12]. Each layer has already been studied, but mostly in isolation. Integrating them creates a more coherent model of why nutraceutical interventions might influence both insulin sensitivity and reproductive cyclicity. It also creates a sharper translational agenda, because the relevant end points are no longer only body weight or testosterone, but also microbial metabolites, inflammatory tone, SIRT1 activity, luteinizing hormone dynamics, and potentially kisspeptin-associated neuroendocrine signatures [13].

## 2. Search Strategy and Selection Criteria

This review employed a structured and iterative literature search strategy to identify relevant studies examining the role of algae-derived bioactives in modulating the gut–SIRT1–kisspeptin axis in PCOS. Electronic databases including PubMed, Scopus, Web of Science, and ScienceDirect were systematically searched for articles published between 2010 and 2026 to capture both foundational and emerging evidence. The search combined controlled vocabulary (e.g., MeSH terms) and free-text keywords using Boolean operators. Core search strings included combinations of “polycystic ovary syndrome” OR “PCOS”, “gut microbiota” OR “dysbiosis”, “SIRT1” OR “AMPK/SIRT1 pathway”, “kisspeptin” OR “GnRH regulation”, and “algae”, “seaweed”, “fucoidan”, “fucoxanthin”, “phlorotannins”, “spirulina”, “chlorella”. Additional filters such as “in vivo”, “clinical trial”, “mechanistic study”, and “review” were applied to refine relevance. Reference lists of selected articles were also manually screened to identify additional pertinent studies not captured in the initial search.

Study selection followed predefined inclusion and exclusion criteria to ensure methodological consistency and relevance to the proposed mechanistic framework. Included studies met the following criteria: (1) original research articles, systematic reviews, or meta-analyses; (2) studies involving human subjects, animal models, or in vitro systems relevant to PCOS, metabolic dysfunction, gut microbiota, SIRT1 signaling, or kisspeptin regulation; and (3) investigations reporting on algae-derived compounds or mechanistically related pathways (e.g., microbiota modulation, AMPK/SIRT1 activation, endocrine regulation). Exclusion criteria comprised (1) non-English publications; (2) conference abstracts without full data; (3) studies lacking mechanistic or clinical relevance to PCOS or the gut–metabolic–neuroendocrine axis; and (4) duplicate or redundant publications.

The screening process was conducted in two stages. First, titles and abstracts were independently assessed to exclude clearly irrelevant studies. Second, full-text articles were reviewed in detail to confirm eligibility and extract key data. Particular emphasis was placed on studies contributing to at least one component of the proposed tri-axis model (gut microbiota, SIRT1 signaling, or kisspeptin/neuroendocrine regulation), as well as those investigating algae-derived interventions. Given the integrative nature of this review, both direct evidence (e.g., algae interventions in PCOS models) and indirect but mechanistically linked evidence (e.g., microbiota–SIRT1 interactions or SIRT1–kisspeptin regulation) were included to construct a comprehensive and translationally relevant framework.

## 3. Gut Dysbiosis in Polycystic Ovary Syndrome

**Figure 2:**
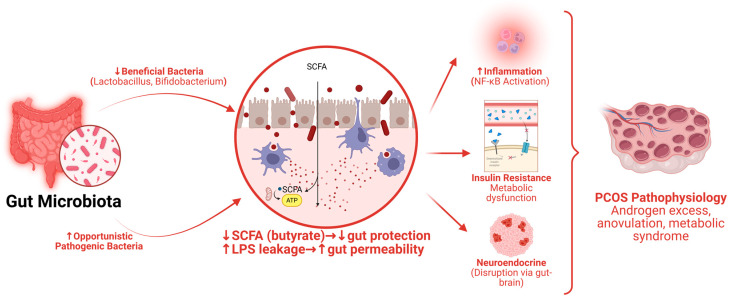
Gut dysbiosis-driven metabolic and inflammatory pathways in PCOS.

The gut microbiota literature in PCOS has evolved from isolated case–control datasets to broader multi-population analyses (Figure 2). The overall pattern is now reasonably consistent, although the specific taxa vary across cohorts. Women with PCOS tend to show lower microbial richness or altered beta diversity. These changes reflect a shift away from a health-associated ecological structure and are accompanied by measurable alterations in the metabolite environment [14]. A 2025 systematic review described a distinct dysbiotic profile in PCOS, while newer cohort synthesis shows that microbial signatures also differ by testosterone status, a crucial clue that microbiota changes may track endocrine subtypes rather than the diagnosis alone [15]. In a 2024 systematic analysis, the higher-testosterone subgroup was enriched for genera such as *Prevotella*, *Blautia*, *Dialister*, and the *Ruminococcus torques* group, whereas *Alistipes*, *Dysosmobacter*, *Phocaeicola*, and *Faecalibacterium* were diminished [16].

This heterogeneity helps explain why single-taxon narratives often fail. Some cohorts report lower abundance of beneficial genera such as *Bifidobacterium*; others do not [17,18]. Some studies highlight *Bacteroides* or *Escherichia-Shigella*; others point to butyrate-associated taxa [15,19]. The more stable message is functional: PCOS-associated dysbiosis appears to favor intestinal permeability, endotoxin exposure, altered steroid and bile acid handling, and disruption of microbial metabolites that communicate with host tissues. Reviews focused on gut dysbiosis in PCOS consistently link these changes to insulin resistance, hyperandrogenism, chronic inflammation, and metabolic dysfunction [20].

One of the most major mechanistic breakthroughs came from the bile acid–interleukin-22 axis. The landmark study by Qi and colleagues showed that *Bacteroides vulgatus* was elevated in individuals with PCOS and accompanied by reduced glycodeoxycholic acid and tauroursodeoxycholic acid [13]. In experimental transfer models, fecal microbiota from women with PCOS, or colonization with *B. vulgatus*, worsened ovarian dysfunction and insulin resistance; conversely, restoration of the downstream bile acid–IL-22 pathway improved the phenotype. This work matters because it moves the microbiome-PCOS link beyond association and into causal metabolic-endocrine signaling. It also provides a mechanistic bridge that is highly relevant to algae, because many algae-derived polysaccharides and polyphenols modulate the very gut microbial and bile acid systems implicated here.

Short-chain fatty acids demand more careful interpretation than is often acknowledged in the PCOS literature. It is tempting to state simply that SCFAs are “reduced” in PCOS, but human data are more nuanced. A 2024 cross-sectional study in women with PCOS found altered microbiota composition together with lower circulating indole-3-propionic acid and higher circulating acetic and propionic acids, while specific fecal SCFA patterns also differed from controls [21]. Another work emphasizes depletion of SCFA-producing taxa and subgroup-specific SCFA disturbances, especially in more metabolically disturbed forms of PCOS [22]. The most defensible conclusion is therefore not uniform SCFA depletion, but a disturbed fermentation landscape in which SCFA production, absorption, compartmentalization, and host utilization are altered. That distinction is vital when designing nutraceutical strategies, because a favorable intervention may need to normalize SCFA flux rather than simply increase absolute SCFA concentrations.

Experimental evidence also suggests that microbial metabolites can influence ovarian tissue directly. Butyric acid has been shown to ameliorate granulosa-cell inflammation and dysfunction in experimental PCOS, supporting the concept that gut-derived fermentative products are not merely systemic metabolic markers but active reproductive modifiers [23]. In parallel, probiotic interventions provide proof-of-concept that changing the intestinal ecology can alter endocrine output. The *Bifidobacterium lactis* V9 study in women with PCOS proposed that probiotic manipulation could regulate sex hormones through a gut–brain route; later reviews summarizing this work note reductions in LH-related measures and improvements in sex-hormone handling [24]. Together, these data support a biologically meaningful gut–ovary and gut–brain–ovary axis in PCOS.

What remains less mature is the direct gut–brain literature in PCOS. A 2025 review of studies on the gut microbiome and mental health in PCOS concluded that the field is still preliminary, with only a small number of human and rodent studies and frequent design limitations [25]. Even so, the direction of evidence is consistent: women with PCOS and mental-health symptoms often show increased Gram-negative bacteria, and reviews of PCOS pathogenesis now routinely discuss microbially derived neurotransmitters, gut peptides, and inflammatory routes that may influence central reproductive control. This does not yet prove a gut-to-kisspeptin pathway in humans with PCOS, but it makes the hypothesis entirely reasonable (Table 1).

## 4. SIRT1 as a Metabolic and Ovarian Integrator

**Figure 3:**
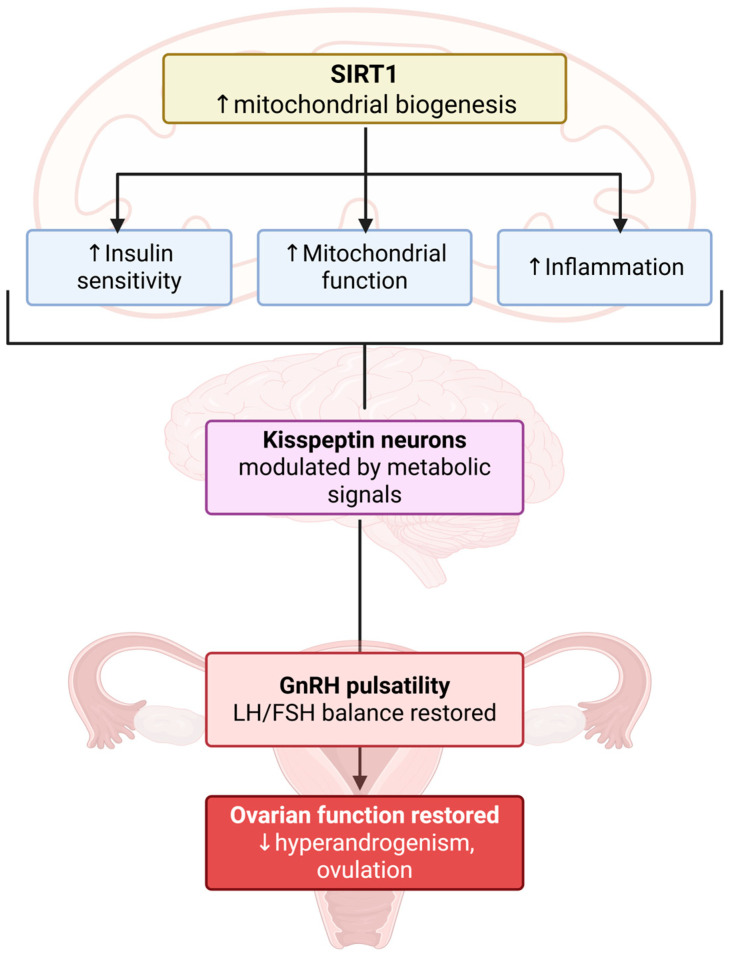
Integration of metabolic and neuroendocrine regulation via the SIRT1–kisspeptin axis.

SIRT1 occupies an attractive position in PCOS pathophysiology because it integrates nutrient state, redox balance, mitochondrial quality control, and inflammatory signaling (Figure 3) [27,28]. Mechanistically, SIRT1 is an NAD^+^-dependent deacetylase; its activity is therefore tightly coupled to cellular energy status. Reviews of the AMPK/SIRT1/PGC-1α axis describe a positive feedback relationship in which AMPK raises NAD^+^ availability and thereby activates SIRT1, while SIRT1 in turn promotes mitochondrial and oxidative programs that reinforce metabolic adaptation [29,30]. This is a hallmark of PCOS, where insulin resistance, oxidative stress, and mitochondrial dysfunction are recurrent features across tissues.

Across ovarian and reproductive compartments, the balance of evidence now favors reduced SIRT1 tone in PCOS. A rat study showed that ovarian SIRT1 expression decreases in PCOS and rises after exenatide or metformin treatment [31]. A 2025 case–control study found reduced SIRT1 protein in the follicular fluid of women with PCOS, alongside reduced aromatase activity and a higher HOMA index [32]. More recently, granulosa-cell analyses identified SIRT1 as downregulated in PCOS-associated ferroptosis and showed that reduced SIRT1 can contribute to impaired follicular cell survival [33]. These studies collectively imply that loss of SIRT1 is not a peripheral epiphenomenon, but part of the microenvironment that shapes follicle quality, steroidogenesis, and ovulatory competence.

The importance of SIRT1 becomes even clearer when examined alongside inflammatory and endometrial phenotypes. In a 2024 study, androgen excess in human endometrial stromal cells reduced the AMPK/SIRT1/PDK4 axis and impaired decidualization, suggesting that defective SIRT1 signaling may help explain not only anovulation but also implantation-related abnormalities in PCOS [34]. Separately, semaglutide improved ovarian inflammation in PCOS mice through the AMPK/SIRT1/NF-κB pathway, reinforcing that SIRT1 sits at a central anti-inflammatory control point rather than at the edge of the disease network [35]. Animal work on autophagy and oxidative stress in letrozole-induced PCOS similarly reports marked reductions in ovarian SIRT1 with parallel defects in LKB1/AMPK signaling [36].

This does not mean that “more SIRT1 is always better.” SIRT1 is a rheostat, not a one-directional switch. In ovarian tissue, restoring deficient SIRT1 appears broadly beneficial because it improves insulin sensitivity, reduces oxidative stress, limits inflammatory signaling, and supports steroidogenic balance [37]. In the hypothalamus, however, SIRT1 can suppress Kiss1 expression when excessively activated in states of energy deficit [38,39]. For PCOS, the therapeutic goal is therefore likely normalization of SIRT1 dynamics in the appropriate tissue compartments, rather than indiscriminate global activation. That nuance is central to a sophisticated algae-based framework, because nutraceuticals rarely act as maximal pharmacological agonists; they more often shift network tone [38].

The gut connection is especially essential here. Because SIRT1 activity is NAD^+^-dependent and AMPK-responsive, it is sensitive to the metabolic consequences of dysbiosis, substrate overload, and systemic inflammation. Reviews on sirtuins and the gut microbiota now frame SIRT1 as both a target and a mediator of microbiome-driven metabolic disease [40]. Outside PCOS, butyrate-rich or microbiota-restoring interventions repeatedly converge on the AMPK/SIRT1/PGC-1α axis. In PCOS specifically, direct proof that microbial metabolites restore SIRT1 in ovarian or hypothalamic tissue remains incomplete, but the mechanistic logic is already strong enough to justify the hypothesis.

## 5. Kisspeptin and Neuroendocrine Dysfunction

Kisspeptin is a central regulator of reproductive neuroendocrine output. Through KISS1/KISS1R signaling, it activates GnRH neurons and shapes the amplitude and frequency of LH release [41]. In health, this system allows nutritional information, steroid feedback, and ovarian-cycle cues to be translated into appropriate pulsatile gonadotropin secretion. In PCOS, however, elevated GnRH pulse frequency and preferential LH synthesis are central elements of hyperandrogenic pathophysiology. Modern reviews of neuroendocrine PCOS increasingly view abnormal hypothalamic signaling not as a downstream response to ovarian dysfunction, but as part of the core disease engine [42,43].

Human kisspeptin data in PCOS are informative but heterogeneous. Meta-analytic and narrative reviews generally report higher circulating kisspeptin levels in PCOS and interpret this as support for an overactive KISS1 system. A 2021 study reported that patients with PCOS had higher kisspeptin, LH, insulin, AMH, and androgen levels together with higher HOMA-IR and lower SHBG [44]. Yet, not all cohorts show elevated kisspeptin, and recent conference or small-study data suggest that some phenotypes may have levels comparable with those of controls. This heterogeneity should not be viewed as a weakness of the hypothesis; it probably reflects the same biological diversity seen elsewhere in PCOS. Hyperandrogenic and oligomenorrheic phenotypes are unlikely to behave identically to milder or more metabolically dominated forms [45].

Perhaps the most revealing data are not the basal hormone levels but the pulse relationships. In a detailed pulsatility study, kisspeptin and LH showed temporal coupling only in eumenorrheic PCOS, whereas in oligomenorrheic PCOS this coupling was lost [46]. Later neuroendocrine review interprets this uncoupling as evidence that the normal kisspeptin-assisted control of GnRH/LH pulsatility is disturbed in more severe disease [47]. Conceptually, this supports a treatment goal broader than lowering testosterone: the more fundamental objective may be to re-stabilize the pulse generator. That makes kisspeptin highly relevant to any integrated mechanistic paper, because it is the point at which metabolic stress becomes reproductive rhythm.

SIRT1 provides the missing metabolic bridge into this system. A pivotal Nature Communications study demonstrated that SIRT1 is expressed in hypothalamic Kiss1 neurons, represses Kiss1 transcription, and functions as an epigenetic conduit for nutritional cues [38]. Overnutrition lowered hypothalamic SIRT1 and accelerated Kiss1 expression, whereas undernutrition increased SIRT1, repressed Kiss1, and delayed puberty. That work has since been extended to adulthood: a 2025 study reported that hypothalamic SIRT1 also regulates the hormonal trigger of ovulation, with central SIRT1 activation attenuating the preovulatory surge and SIRT1 inhibition augmenting it [39]. These findings are relevant for PCOS because they imply that abnormal energy sensing can directly distort kisspeptin-dependent reproductive output.

The implication is subtle but crucial. In PCOS, the desired endpoint is not simple suppression of kisspeptin; it is restoration of physiological tuning. If hypothalamic SIRT1 is too low in an overnourished, hyperandrogenic state, kisspeptin drive may become excessive or temporally disorganized. Restoring more normal SIRT1-dependent regulation could help recalibrate rather than abolish the reproductive axis. Emerging ovarian data make kisspeptin even more interesting: a 2026 study reported that kisspeptin improved local ovarian insulin resistance through PI3K/AKT/GLUT4 signaling and mitochondrial protection in granulosa cells. That observation suggests that kisspeptin may not be purely central in PCOS but may also participate in intra-ovarian metabolic repair [48].

The gut–brain component remains the least mature but perhaps the most innovative. Gut dysbiosis can modify neurotransmitter precursors, immune mediators, bile acid receptors, and vagal signaling. Reviews of the gut–brain axis in PCOS now acknowledge this route, but also emphasize its methodological immaturity [49,50]. Still, the *Bifidobacterium lactis* V9 study, which linked microbiota manipulation to sex-hormone shifts through a gut–brain framework, demonstrates that microbiota-to-endocrine signaling in PCOS is more than speculation [24]. For a review paper, the appropriately rigorous position is that the gut–kisspeptin route is plausible and partly supported but not yet proven at the pathway level in humans.

## 6. Algae-Derived Bioactives as Multi-Target Modulators

### 6.1. Bioactivity and Multi-Target Mechanisms of Algae-Derived Compounds

Algae are unusually well-suited for multi-system disorders because they combine poorly absorbed colon-active fractions with absorbable system-active molecules. Seaweed polysaccharides can reach the large intestine, where they act as fermentation substrates or ecological modulators. Phlorotannins undergo extensive microbial transformation. Fucoxanthin and related carotenoids influence redox and lipid pathways. In addition, microalgae biomass provides proteins, pigments, trace elements, and cell-wall components that can modulate host metabolism (Table 2) [51,52]. Thus, algae offer a rare combination of prebiotic-like, anti-inflammatory, antioxidant, and endocrine-relevant activity in a single nutritional domain [53].

The evidence for seaweed polysaccharides and gut modulation is now strong. A 2026 review in Carbohydrate Polymers summarized that low-molecular-weight algae polysaccharides are more readily fermented by gut bacteria, and that structural features such as sulfation and carboxyl groups influence SCFA output and taxonomic shifts [53]. Agar derivatives, porphyran, fucoidan, alginate, laminarin, ulvan, and sulfated rhamnans all showed microbiota-modifying effects, with several increasing SCFA production and some lowering the *Firmicutes*/*Bacteroidetes* ratio or enriching *Bacteroidetes* [54,55]. The translational implication is straightforward: algal structure is not a manufacturing detail but a biological determinant of microbiome effect size.

Phlorotannins are especially attractive in a gut-centered PCOS framework because their low oral bioaccessibility actually becomes an advantage. Rather than being fully absorbed proximally, they enter the colon and interact with the microbiota. In vitro fermentation work with *Silvetia compressa* showed that phlorotannin-rich hydroethanolic extract promoted *Bifidobacterium* and *Lactobacillus* growth and increased total SCFA production to levels comparable with inulin [56]. Complementary work with *Fucus vesiculosus* phlorotannins showed enhancement of propionate and butyrate generation, despite limited upper-gut bioaccessibility [57]. Recent synthesis in Frontiers in Nutrition further argues that phlorotannins are poorly absorbed in the upper gut, depend heavily on microbial transformation, can promote beneficial bacteria, and may regulate metabolism partly through SCFAs, AMPK, PPAR signaling, and bile-acid-related pathways [58].

Fucoidan and fucoxanthin add a second mechanistic layer because they bridge gut effects and intracellular metabolic signaling. Fucoidan has repeatedly been shown in non-PCOS metabolic models to improve steatosis and inflammation through AMPKα1/SIRT1-related pathways while simultaneously reshaping the gut microbiota and bile acid axis [59,60]. One 2021 study reported that fucoidan inhibited alcohol-induced steatosis and bile acid disorders through the AMPKα1/SIRT1 pathway and the gut microbiota–bile acid–liver axis [61]. Earlier work with low-molecular-weight fucoidan demonstrated protection against fatty liver through SIRT1/AMPK/PGC-1α signaling [62]. Fucoxanthin, meanwhile, has been reported to activate SIRT1/Nrf2/HO-1 signaling and, in high-fat-fed mice, to ameliorate obesity alongside modulation of bile acid metabolism and gut microbiota [63]. In vitro fermentation models also show that fucoxanthin can reshape microbiota composition and metabolic output even in non-obese individuals [64].

Direct PCOS models, although still mostly preclinical, are encouraging. *Ecklonia cava* extract restored estrous cyclicity; normalized testosterone, estrogen, LH, FSH, and AMH; improved ovarian histology; and increased Cyp19a1 and estrogen-receptor-related expression in letrozole-induced PCOS rats [65]. A follow-up study combining *Ecklonia cava* with D-chiro-inositol also reduced LH, testosterone, and inflammatory cytokines [66]. In DHEA-induced PCOS rats, *Spirulina platensis* improved the expression of genes linked to androgen signaling, steroidogenesis, apoptosis, and metabolic control; follow-up work comparing spirulina with metformin reported improvement in insulin-signaling and glucose-homeostasis genes [67]. A 2025 mouse study reported that *Chlorella vulgaris* reduced testosterone and LH, improved ovarian morphology and oxidative-stress markers, and restored the disturbed gut microbiota [68]. Astaxanthin, a microalgal carotenoid, has also shown protective effects in experimental PCOS ovaries and livers [69].

Human data are more limited and more mixed, which is exactly what a serious review should acknowledge. The strongest human fucoxanthin signal comes from a randomized placebo-controlled trial in metabolic syndrome, in which 12 mg/day for 12 weeks was tested for effects on insulin sensitivity and secretion [70]. By contrast, a 2024 study of microalgae extract from *Phaeodactylum tricornutum* containing 4.4 mg/day fucoxanthin reported no additional weight or fat loss in overweight women over 12 weeks [71]. Spirulina has stronger human metabolic support overall: randomized trials and systematic reviews report improvement in insulin sensitivity, lipid profile, antioxidant status, and metabolic-syndrome components. None of these studies were conducted specifically in women with PCOS, but they are relevant because insulin resistance and low-grade inflammation are central to the syndrome.

**Table 2:** Algae-derived bioactives relevant to the gut–SIRT1–kisspeptin framework.

Algal Source	Main Bioactives	Dominant Gut Action	SIRT1-Related or Metabolic Action	PCOS-Relevant Signal	Key Evidence
Brown seaweeds	Fucoidan, alginate, laminarin	Fermentation substrate; bile-acid and microbial restructuring	AMPK/SIRT1/PGC-1α support	Metabolic inflammation, insulin resistance, bile-acid dysregulation	[53]
Brown seaweeds	Phlorotannins	↑ *Bifidobacterium*/*Lactobacillus*; ↑ propionate/butyrate	AMPK, PPAR, antioxidant signaling	Gut barrier and metabolic repair	[56]
Brown algae and microalgae	Fucoxanthin	Gut microbiota and bile acid modulation	SIRT1/Nrf2/HO-1; anti-obesity and anti-diabetic actions	Indirect relevance to IR-dominant PCOS	[63]
Brown algae	*Ecklonia cava* extract	Not primarily microbiota-studied in the PCOS model	Steroidogenic and anti-inflammatory effects	Restored estrous cycle and hormone profile in PCOS rats	[65]
Green microalgae	*Chlorella vulgaris*	Gut microbiota restoration	Antioxidant and steroid-hormone rebalancing	Reduced testosterone and LH; improved ovarian morphology in PCOS mice	[68]
Cyanobacterial biomass	*Spirulina platensis*/*maxima*	Gut permeability and microbiota modulation	Insulin sensitivity and antioxidant effects	Improved PCOS gene signatures in rats; metabolic benefits in humans	[72]

### 6.2. Gastrointestinal Biotransformation of Algae-Derived Bioactives

The physiological effects of algae-derived compounds are strongly influenced by their chemical transformation within the gastrointestinal tract. Many of these bioactives exhibit limited bioaccessibility in the upper intestine and therefore undergo extensive microbial biotransformation in the colon, resulting in structurally modified metabolites with distinct biological activities.

For example, phlorotannins, which are high-molecular-weight polyphenols composed of phloroglucinol units, are poorly absorbed in their native form. In the colon, they are subjected to microbial enzymatic processes, including depolymerization, dehydroxylation, and ring cleavage, generating lower-molecular-weight phenolic acids such as hydroxybenzoic acid and hydroxyphenylacetic derivatives [73]. These metabolites exhibit increased bioavailability and can modulate host signaling pathways, including AMPK activation and inflammatory regulation.

Similarly, fucoidan, a sulfated polysaccharide rich in fucose residues, is resistant to human digestive enzymes but can be partially degraded by gut microbial enzymes such as fucoidanases and glycosidases. This process yields oligosaccharides and desulfated fragments that influence microbial composition and may interact with host receptors involved in immune and metabolic regulation [74]. Structural features such as molecular weight, degree of sulfation, and branching pattern critically determine its fermentability and downstream biological effects.

Fucoxanthin undergoes a different transformation pathway. After ingestion, it is hydrolyzed in the gastrointestinal tract to fucoxanthinol and subsequently converted in the liver to amarouciaxanthin A [75]. These metabolites exhibit greater systemic bioactivity than the parent compound and have been associated with modulation of lipid metabolism, oxidative stress, and insulin signaling pathways.

Collectively, these examples illustrate that the biological activity of algae-derived compounds is not solely determined by their native structures, but also by their biotransformation into secondary metabolites. This transformation process provides a mechanistic link between gut microbial activity and host metabolic signaling, reinforcing the relevance of the gut–SIRT1–kisspeptin axis proposed in this review (Table 3).

## 7. An Integrated Gut–SIRT1–Kisspeptin Framework for PCOS

**Figure 4:**
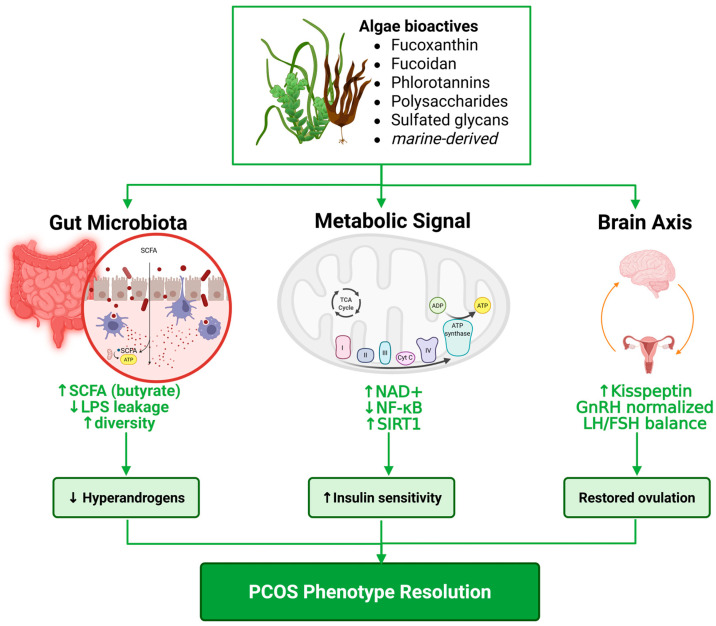
Algae-derived bioactives reprogram the gut–SIRT1–kisspeptin axis in PCOS.

The strongest version of the proposed mechanism begins in the intestine. Many algae-derived polysaccharides and polyphenols are only partially digested or absorbed in the upper gastrointestinal tract, which allows them to arrive in the colon, where they can reshape substrate availability and microbial competition (Figure 4). In that environment, phlorotannins and seaweed polysaccharides can enrich beneficial bacteria, alter the *Firmicutes*/*Bacteroidetes* balance, and increase production of propionate, butyrate, and other fermentation products [76]. In other words, algae are especially suited to intervene at the precise site where the PCOS microbiome is disturbed.

The second step is improvement of microbial signaling quality. In PCOS, dysbiosis is linked to altered bile acids, low-grade inflammation, and disturbed microbial metabolite profiles [77]. If algae-derived interventions shift the microbiota toward a more favorable ecological state, then several downstream consequences become plausible: lower LPS burden, restoration of bile-acid pools that influence FXR/TGR5 and IL-22 signaling, and normalization of SCFA and indole-derived metabolites [78]. That logic is reinforced by the Qi study showing a causal *Bacteroides vulgatus*–bile acid–IL-22 route in PCOS, and by human cross-sectional evidence linking PCOS diagnosis to lower indole-3-propionic acid and altered circulating SCFAs [13].

The third step is convergence on AMPK/NAD^+^/SIRT1. This is the biological hinge of the model. SIRT1 is an NAD^+^-dependent energy sensor, AMPK can raise NAD^+^ and activate SIRT1, and butyrate-rich microbiota-modifying interventions often restore the AMPK/SIRT1/PGC-1α axis in metabolic disease. At the same time, several algae-derived compounds themselves, notably fucoidan and fucoxanthin, have already been shown to activate SIRT1-related signaling in non-PCOS metabolic injury models [79]. Thus, algae may engage SIRT1 twice: indirectly by improving the metabolite and inflammatory environment generated by the gut, and directly by influencing intracellular redox and deacetylase pathways. This dual engagement is one reason algae are more conceptually attractive than isolated probiotic strains for a multi-axis disorder [29].

The fourth step is tissue divergence. In the ovary and endometrium, restored SIRT1 would be expected to reduce oxidative stress, limit inflammatory and ferroptotic injury, improve steroidogenic balance, and support insulin-sensitive cellular function [80]. In the hypothalamus, the same metabolic improvement could re-tune Kiss1 control of GnRH/LH pulsatility [81]. Importantly, the argument is not that algae should maximally activate hypothalamic SIRT1; that could oversuppress reproductive signaling. Rather, by shifting the organism away from the metabolic-inflammatory state that disturbs hypothalamic energy sensing, algae could plausibly normalize the SIRT1–Kiss1 relationship. This is consistent with the known bidirectionality of SIRT1 action in reproductive neuroendocrinology [32].

The fifth step is phenotypic expression. If gut dysbiosis, inflammatory tone, and SIRT1 dysfunction are simultaneously improved, then the measurable outputs should include lower androgen excess, better insulin sensitivity, improved follicular microenvironment, and more regular ovulatory cycling. That is exactly the pattern already described in the best algae-PCOS animal studies, especially those using *Ecklonia cava*, spirulina, and chlorella [65]. These studies do not yet prove the full gut–SIRT1–kisspeptin route, but they do show the type of downstream phenotype that such a route would predict.

For publication-quality scholarship, it is important to distinguish strong from tentative links. Strong links include PCOS-to-dysbiosis, dysbiosis-to-bile acid signaling, SIRT1 deficiency in reproductive tissues, hypothalamic SIRT1 control of Kiss1, and algae-to-gut or algae-to-SIRT1 signaling [20]. Moderate links include microbiota-to-sex-hormone modulation and microbiota-to-gut–brain endocrine coupling in PCOS. Tentative links include the full algae, microbiota, SIRT1, kisspeptin, and PCOS correction chains in a single model. That full chain is precisely the novelty of the present framework, but it remains a framework rather than a fully validated pathway. A high-impact review should make that distinction explicit, because doing so strengthens rather than weakens the argument.

Another crucial observation is what the algae-PCOS literature has not measured. The direct PCOS intervention studies identified for this review assessed endocrine profiles, ovarian morphology, inflammatory markers, oxidative stress, or insulin-signaling genes. The chlorella work added microbiota profiling. None of these studies directly examined hypothalamic kisspeptin or SIRT1-driven reproductive pulse generation. This is the clearest translational gap and also the clearest opportunity: a future study that measures gut metabolites, tissue SIRT1, and kisspeptin-related neuroendocrine outputs in the same experimental design would not be incremental; it would materially move the field.

## 8. Translational and Practical Considerations for Algae-Based Interventions

### 8.1. Translational Outlook and Future Directions

The translational attraction of algae for PCOS lies in their alignment with three current priorities in modern endocrine therapeutics: microbiome-aware intervention, precision nutrition, and low-toxicity long-term management (Table 4). Unlike standard pharmaceuticals, algae-based products can be developed as functional foods, supplements, or purified nutraceuticals; unlike generic functional foods, they already carry mechanistic relevance to the PCOS hallmarks of inflammation, insulin resistance, oxidative stress, and endocrine dysregulation. However, the field is not yet ready for broad clinical claims because almost all direct PCOS evidence remains preclinical. Human data support metabolic plausibility, not disease-specific efficacy.

The next challenge is formulation. Not all algae preparations are biologically equivalent. For fucoidan, molecular weight and sulfation pattern influence fermentability and host response. For phlorotannins, upper-gut bioaccessibility is low, and therefore microbial transformation becomes central to efficacy. For fucoxanthin, absorption and tissue delivery are major limiting factors, which is one reason human trials sometimes show weaker signals than rodent studies. Even whole-biomass products such as spirulina or chlorella can vary substantially in pigment content, cell-wall digestibility, contaminant profile, and accompanying micronutrients. A serious translational agenda therefore has to move beyond botanical naming and towards chemical standardization.

Clinical trial design should also become phenotype-aware. The older habit of pooling all PCOS phenotypes into one nutraceutical trial is increasingly hard to justify. Hyperandrogenic, obesity-associated, insulin-resistant, and normal-weight phenotypes likely differ in microbiota architecture, metabolite profile, and neuroendocrine disturbance. Recent microbiota analyses already show that testosterone level stratifies the intestinal signature [82]. That means algae interventions might work best in subgroups enriched for dysbiosis-driven metabolic inflammation, while other subgroups may require combination therapy. Precision nutrition in PCOS should therefore include not only baseline BMI and HOMA-IR but also microbial composition, bile acid profile, and perhaps reproductive-hormone pulsatility where feasible.

Biomarker selection is where this field could become truly original. A future mechanistic trial of algae in PCOS should not stop at testosterone and irregular cycles. The most informative design would combine stool metagenomics or 16S profiling, fecal and plasma SCFAs, bile acid metabolomics, serum inflammatory mediators, insulin sensitivity measures, and tissue-specific SIRT1 readouts where accessible [83]. In IVF populations, follicular-fluid SIRT1 is now measurable and already linked to PCOS pathogenesis. On the neuroendocrine side, serum kisspeptin alone may be too noisy, but LH pulsatility, kisspeptin-LH temporal relationships, or other proxy markers of GnRH pulse control could provide deeper mechanistic resolution. Even small studies would be highly informative if they used rich phenotyping.

Safety and implementation also deserve sober attention. Brown algae can contain substantial iodine, which may be relevant for patients with thyroid risk; reviews of phlorotannin supplementation emphasize that no direct toxic effects of phlorotannins themselves have been established, but extract composition matters. Whole-biomass microalgae require quality control for heavy metals, microbial contamination, and batch consistency. In addition, reproductive-stage women may need specific guidance during conception attempts and pregnancy, where the general PCOS guideline still prioritizes evidence-based reproductive and metabolic care over unproven adjuncts. In other words, algae should presently be positioned as rational adjunct candidates rather than replacements for standard therapy [58].

The most defensible final conclusion is, therefore, twofold. First, the mechanistic landscape already supports a compelling translational concept: algae-derived bioactives can influence the gut ecosystem, intersect with SIRT1-centered metabolic signaling, and plausibly reshape neuroendocrine dysfunction relevant to PCOS. Second, the decisive experiment has not yet been done. The field now needs integrated studies that measure gut dysbiosis, microbial metabolites, SIRT1 activity, kisspeptin-related neuroendocrine outputs, and reproductive phenotype in the same model or trial. If that evidence emerges, algae would move from being an interesting adjunct in PCOS to being a genuine systems-level therapeutic strategy. Until then, the gut–SIRT1–kisspeptin axis is best understood as a high-value mechanistic framework that organizes scattered observations into a coherent, testable, and clinically meaningful theory.

**Table 4:** Representative intervention studies that inform the algae–PCOS translational pipeline.

Intervention	Context	Main Readouts	Main Outcome	Axis Nodes Touched	Key Evidence
*Ecklonia cava* extract	Letrozole-induced PCOS rats	Estrous cycle, testosterone, E2, LH, FSH, AMH, ovarian histology, Cyp19a1/ER pathways	Restored cycle, hormones, ovarian morphology	Ovarian steroidogenesis; inflammation	[65]
*Spirulina platensis*	DHEA-PCOS rats; comparison with metformin	AR/CYP19A1/HSD3B1/SRD5A1/BCL2/BAX; hepatic insulin-signaling genes	Improved PCOS-related endocrine and metabolic gene signatures	Insulin signaling; oxidative stress; ovary	[84]
*Chlorella vulgaris*	DHEA-PCOS mice	Testosterone, LH, ovarian morphology, oxidative-stress genes, gut microbiota	Reduced testosterone/LH, improved morphology, restored dysbiosis	Gut–ovary axis	[68]
Astaxanthin	Experimental PCOS rats	Ovarian and hepatic oxidative-stress/histology readouts	Protective effects in ovary and liver	Oxidative stress; metabolic injury	[85]
Fucoxanthin (12 mg/day)	Adults with metabolic syndrome	Insulin sensitivity and secretion	Human proof-of-concept for metabolic benefit	Metabolic arm of PCOS	[70]
*Phaeodactylum tricornutum* extract (4.4 mg/day fucoxanthin)	Overweight women, 12 weeks	Body composition	No additional weight/fat loss	Translational caution on dose and population	[71]

### 8.2. Dose Considerations, Safety, and Comparison with Conventional Plant-Derived Interventions

The physiological effects of algae-derived bioactives are strongly influenced by dose, formulation, and bioavailability. However, dose–response relationships remain incompletely characterized, particularly in PCOS-specific contexts. Available human studies suggest that effective doses vary substantially across compounds. For example, fucoxanthin has been evaluated at approximately 4–12 mg/day in human metabolic studies, with mixed outcomes depending on formulation and population characteristics [70]. Spirulina supplementation is more extensively studied, with typical doses ranging from 1 to 5 g/day, showing improvements in insulin sensitivity, lipid profile, and inflammatory markers in metabolic disorders [86]. For fucoidan, reported human doses vary widely (e.g., 100–1000 mg/day), reflecting differences in molecular weight and sulfation patterns that influence bioactivity [87]. These variations highlight that biological effects cannot be generalized across algal products without consideration of chemical composition and delivery form (Table 5).

Safety and tolerability also require careful consideration. While algae-derived compounds are generally regarded as safe in moderate doses, potential adverse effects have been reported. Brown seaweeds may contain high levels of iodine, which can affect thyroid function, particularly with excessive intake or in susceptible individuals. Contamination with heavy metals (e.g., arsenic, cadmium) and environmental toxins represents an additional concern for poorly standardized products. Gastrointestinal discomfort, including bloating or diarrhea, has been reported with high intake of polysaccharide-rich preparations [76]. For microalgae such as Spirulina and Chlorella, quality control is critical to avoid contamination with microcystins or pathogenic microorganisms. These considerations emphasize the importance of standardization, purification, and dose optimization in translational applications.

A further important perspective is how algae-derived interventions compare with more conventional plant-based products. Many plant-derived fibers and polyphenols, such as inulin, resistant starch, and flavonoids, also modulate gut microbiota composition and increase short-chain fatty acid production. In this context, algae-derived polysaccharides and polyphenols do not represent entirely unique mechanisms but rather structurally distinct variants of broader dietary bioactives. Their potential advantage lies in specific structural features, such as sulfation in fucoidan or the unique polymerization patterns of phlorotannins, which may confer differential fermentability, microbial selectivity, and downstream signaling effects. However, direct comparative studies between algae-derived compounds and conventional plant-based interventions remain limited. Therefore, current evidence supports the positioning of algae as complementary rather than superior modulators within the broader landscape of microbiome-targeted nutritional strategies.

## Figures and Tables

**Table 1:** Multi-axis disturbances that position PCOS as a gut–metabolic–neuroendocrine disorder.

Node	Typical Alteration in PCOS	Representative Readouts	Functional Implication	Key Evidence
Gut microbial structure	Lower richness in many cohorts; phenotype-specific dysbiosis	Reduced diversity; high-testosterone subgroup enriched *Prevotella*, *Blautia*, *Dialister*; depleted *Alistipes* and *Faecalibacterium*	Links to hyperandrogenism, insulin resistance, inflammation	[16]
Barrier and endotoxin tone	Greater gut permeability and inflammatory signaling	Gram-negative enrichment, LPS-related inflammation, altered barrier defense	Chronic low-grade inflammation; worsened insulin signaling	[26]
Bile acid signaling	Disordered bile acid profile	Reduced glycodeoxycholic acid and tauroursodeoxycholic acid; reduced IL-22 axis tone	Ovarian dysfunction and insulin resistance	[13]
Fermentation metabolites	Disturbed SCFA and tryptophan-metabolite landscape	Higher circulating acetate/propionate in one cohort; lower IPA; subgroup-specific SCFA depletion in others	Altered energy sensing, immune tone, ovarian-cell function	[21]
Gut-to-endocrine signaling	Microbiome changes can shift reproductive hormones	Probiotic V9 altered sex-hormone outputs through a gut–brain mechanism	Supports microbiota-targeted endocrine modulation	[24]

**Table 3:** Gastrointestinal biotransformation of key algae-derived bioactives and their functional implications.

Parent Compound	Structural Class	Major Gastrointestinal Transformation	Representative Metabolites	Functional Effects to PCOS	Key Evidence
Phlorotannins (e.g., eckol, dieckol)	Polyphenols (phloroglucinol polymers)	Microbial depolymerization, dehydroxylation, ring cleavage	Hydroxyphenylacetic acids, hydroxybenzoic acids	Increased bioavailability; modulation of AMPK signaling; anti-inflammatory effects; improvement of gut barrier function	[56,58]
Fucoidan	Sulfated polysaccharide (fucose-rich)	Partial microbial degradation via glycosidases and fucoidanases; desulfation	Fuco-oligosaccharides, desulfated fragments	Modulation of gut microbiota composition; bile acid signaling; immune regulation; indirect activation of AMPK/SIRT1 pathways	[59,61]
Alginate	Anionic polysaccharide (mannuronic/guluronic acids)	Fermentation by gut microbiota	Short-chain fatty acids (acetate, propionate, butyrate)	Improved insulin sensitivity; reduced inflammation; enhancement of gut barrier integrity	[54,55]
Laminarin	β-glucan polysaccharide	Microbial fermentation	SCFAs (especially butyrate)	Regulation of energy metabolism; anti-inflammatory effects; support of intestinal homeostasis	[53,54]
Fucoxanthin	Carotenoid (xanthophyll)	Enzymatic hydrolysis in the intestine; hepatic conversion	Fucoxanthinol, amarouciaxanthin A	Improved lipid metabolism; antioxidant activity; activation of SIRT1/Nrf2 pathways; improved insulin sensitivity	[63,64]
Astaxanthin	Carotenoid	Limited microbial transformation; absorption and systemic distribution	Minor oxidative metabolites	Antioxidant protection; reduction in oxidative stress; improvement of mitochondrial function	[69]
Chlorella-derived polysaccharides	Complex polysaccharides	Microbial fermentation	SCFAs; oligosaccharides	Modulation of gut microbiota; improvement of metabolic homeostasis; reduction in systemic inflammation	[10,68]
Spirulina-derived compounds (e.g., phycocyanin-associated polysaccharides)	Protein-polysaccharide complexes	Partial digestion and microbial fermentation	Bioactive peptides; SCFAs	Anti-inflammatory effects; improved insulin signaling; modulation of gut permeability	[67,72]

**Table 5:** Dose ranges, safety considerations, and comparison with conventional plant-derived bioactives.

Compound	Typical Human Dose Range	Reported Benefits	Safety Considerations	Comparison with Plant-Based Analogs	Key References (DOI)
Fucoxanthin	~4–12 mg/day	Improved insulin sensitivity, lipid metabolism	Limited bioavailability; inconsistent human outcomes	Comparable to carotenoids/polyphenols but with distinct metabolic targets	[70,71]
Fucoidan	~100–1000 mg/day	Anti-inflammatory, metabolic regulation	Iodine co-exposure; variability in purity	Functionally similar to soluble fibers but structurally sulfated	[59,61]
Spirulina	~1–5 g/day	Improved glucose metabolism, antioxidant status	Contamination risk (microcystins); GI discomfort	Comparable to plant protein + antioxidant sources	[67,72]
Chlorella	~2–10 g/day	Lipid lowering, antioxidant effects	Heavy metal contamination risk	Similar to fiber-rich plant supplements	[10,68]
Phlorotannins	Not well standardized	Gut microbiota modulation, anti-inflammatory effects	Limited human dose data	Comparable to terrestrial polyphenols (e.g., flavonoids)	[56,58]
Alginate/Laminarin	Variable (dietary intake)	SCFA production, gut barrier support	GI bloating at high intake	Similar to inulin, pectin, resistant starch	[53,55]

## Data Availability

No new data were created or analyzed in this study. Data sharing is not applicable to this article.
